# Multiple-System Atrophy with Cerebellar Predominance Presenting as Respiratory Insufficiency and Vocal Cords Paralysis

**DOI:** 10.1155/2010/351239

**Published:** 2010-09-01

**Authors:** Ramon Andrade Bezerra de Mello, Diana Ferreira, José Manuel Dias da Costa, Maria José Rosas, João Manuel Quinaz

**Affiliations:** ^1^Department of Internal Medicine, Hospital São João, EPE, 4200-319 Porto, Portugal; ^2^Department of Medicine, School of Medicine, Porto University, 4200-319 Porto, Portugal; ^3^Department of Neurorradiology, Hospital São João, EPE, 4200-319 Porto, Portugal; ^4^Department of Neurology, Hospital São João, EPE, 4200-319 Porto, Portugal

## Abstract

*Background*. MSA (Multiple System Atrophy) may be associated either with Parkinsonism or with cerebellar ataxia (MSA-c subtype). It is considered a rare disease, but many patients are misdiagnosed as suffering from idiopathic Parkinson's disease. In this paper, we report a case of a patient admitted with respiratory failure and vocal cords paralysis due to MSA-c. *Case Report*. A 79-year-old Caucasian woman was admitted in March 2010 with dyspnea, asthenia, stridor, and respiratory failure needing noninvasive ventilation. She had orthostatic blood pressure decline, constipation, insomnia, daytime sleepiness, and snoring. The neurologic examination revealed cerebellar ataxia. A laryngoscopy revealed vocal cord paralysis in midline position and tracheostomy was performed. The Brain Magnetic Resonance Imaging revealed atrophy of middle cerebellar peduncles and pons with the “hot cross bun sign.” *Conclusion*. Although Multiple-system atrophy is a rare disease, unexplained respiratory failure, bilateral vocal cord paralysis, or stridor should lead to consider MSA as diagnosis.

## 1. Introduction

Multiple-system atrophy (MSA) is a rare and neurodegenerative disease. Clinical features include a progressive onset at age 30 or later with autonomic dysfunction, orthostatic decrease of blood pressure, and cerebellar syndrome, which can be frequently misdiagnosed as idiopathic Parkinson's disease. The mean age at onset is about 53 (36–74) years [1, 2]. Between 4% and 22% of the brains in Parkinsonian brain banks presented MSA at postmortem autopsy. The estimated annual incidence of MSA is about 0–6 per 100000 per year, reaching 3 per 100000 per year in the population older than 50. Prevalence ranges from 1–9 to 4–9 per 100000 inhabitants [3]. This disease has a poor prognosis with average overall survival of about five (range 1–11) years [1–4]. A study of the European population revealed predominance of Parkinsonian features in 58% of patients (MSA-P subtype) or cerebellar ataxia in about 42% of patients (MSA-C subtype). Another North American cohort study indicated a similar predominance of the Parkinsonian subtype (60% of MSA-P subtype) and 13% of MSA-C subtype [3]. The second consensus statement on MSA retains the diagnostic categories of MSA-P and MSA-C that are dependent on the predominant presence of motor symptoms during evaluation, and also retains the designations of “possible,” “probable,” and “definite” MSA. The definite diagnosis is based on neuropathological evidence of glial cytoplasmic inclusions (GCIs) in association with striatonigral degeneration or olivopontocerebellar ataxia [3, 4]. The diagnosis of MSA is based on clinical criteria and can be supported by neuroimaging [5]. For possible diagnosis of MSA-c the patient should have Parkinsonism or cerebellar syndrome (gait ataxia with cerebellar dysarthria, limb ataxia or cerebellar oculomotor dysfunction), another feature suggesting autonomic dysfunction and at least one of the following additional criteria: atrophy on resonance imaging of the putamen, middle cerebellar peduncle, or pons, hypometabolism on 18F-2-fluoro-deoxy-D-glucose by photon emission computer tomography (DG-PET) in putamen or presynaptic nigrostriatal dopaminergic denervation on single photon emission computer tomography (SPECT) or PET [3, 6]. The aim of this paper is to report a case of a patient that has been diagnosed with multiple-system atrophy with cerebellar predominance after presenting respiratory failure due to vocal cords paralysis.

## 2. Case Report

**Figure 1:**
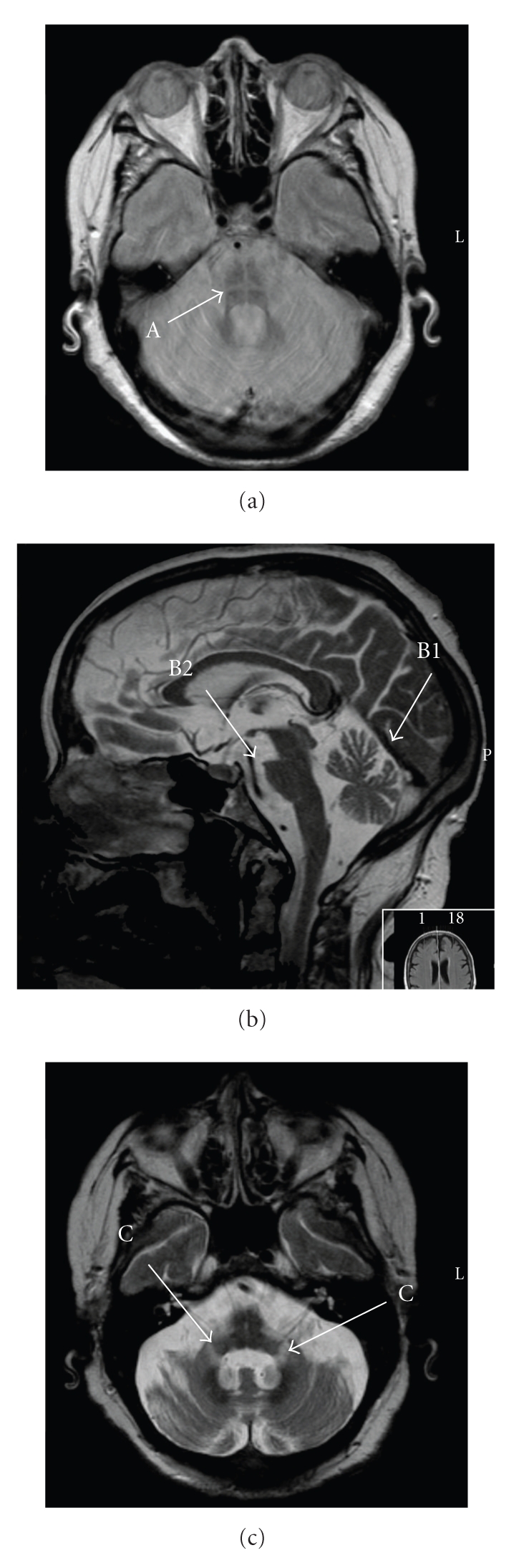
MRI imaging's showing the reported alterations. (A) Hot cross sign. (B1) cerebellar and (B2) pons atrophy. (C) Atrophy of middle cerebellum peduncles.

**Figure 2:**
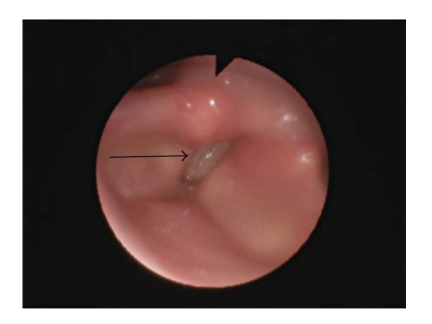
Vocal cords paralysis reported in endoscopy.

A 79-year-old Caucasian woman, single, retired school teacher, with clinical history of systemic arterial hypertension, heart failure, snoring, hip prosthesis placed in 2006, congenital rubeola, lack of the left arm, sinusal bradycardia, and with no relevant familiar history, was admitted in March 2010 complaining of gait ataxia that had persisted for 3 weeks, followed by dyspnea, unconsciousness for few minutes, periods of myotonia in the right arm, stridor, hypercapnia, respiratory acidosis (pH = 7.29, pO_2_ = 111 mmHg, pCO_2_ = 60 mmHg, HCO_3_ = 28), and bradypnea. She was admitted in Intensive Unit Care and began noninvasive ventilation treatment with good clinical response. In order to exclude epilepsy, it was performed an electroencephalogram and brain computed axial tomography (TAC) none of which reported alterations. She was observed in Otolaryngology to study the stridor etiologies that were diagnosed by endoscopy vocal cords diminution of motility and paresis periods in midline position. To exclude sleep disorders, functional respiratory and polysomnographic sleep studies were performed and revealed restrictive pattern by spirometric parameters with Forced Vital Capacity (FVC) = 46%, (Forced Expired Volume in first second (FEV1) = 50%, Tiffeneau index (FEV1/FVC) = 89% and severe sleep apnea syndrome with apnea-hypopnea index = 50.2/h, 16.6% hypopnea events, 83.4% of obstructive apnea events and none central or mixed events. After stabilization of the respiratory status, the patient was transferred to the Internal Medicine ward so that a study of other etiological causes of respiratory failure could be performed. In this evaluation, the patient presented orthostatic arterial hypotension and stridor had worsened. Periods of unconsciousness also followed, as reported by resident physicians. Nasal endoscopy was again performed and as result vocal cord paralysis was revealed in midline. In order to discard other etiologies such as thoracic neoplasm involving the laryngeal recurrent nerve, the patient was observed in Pneumology where a thoracic TAC and a bronchoscopy were performed. No alterations were reported. During the follow-up the patient had other crisis of stridor, agitation, tachypnea, respiratory insufficiency, and respiratory acidosis requiring again noninvasive ventilation, otolaryngological evaluation, and subsequent tracheostomy in the Intensive Care Unit. After stabilization she was again brought back to the Internal Medicine ward. In order to screen the Central Nervous System for disorders like ischemic disease, a Brain Magnetic Resonance Imaging was performed and disclosed atrophy of middle cerebellar peduncles and pons with the “hot cross bun sign” (Figure 1). Imaging findings associated with the presence of the clinical criteria like cerebellar ataxia, limb ataxia, orthostatic blood pressure decline, and stridor are compatible with Multiple-System Atrophy with cerebellar predominance (MSA-c). A new reasessement in Otolaryngology still disclosed vocal cords paralysis (Figure 2) and tracheostomy was kept permanently. The patient was discharged with stable clinical status.

## 3. Discussion

MSA is a rare disease, difficult to diagnose, and presenting motor and autonomic deficits. It was first described by Gram and Oppenheimer in 1969 and redefined in the late 1990s as a progressive neurodegenerative disease of undetermined cause, occurring sporadically and causing Parkinsonism, cerebellar, pyramidal, autonomic, and urological dysfunction, in any combination. The postmortem presence of glial cytoplasmic inclusions is characteristic [3]. In 2004, Cormican et al. reported [7] that multiple-system atrophy should be considered in the differential diagnosis of late onset central sleep apnea and progressive hypoventilation. Vocal cord paralysis is unusual in MSA, but must be taken into consideration. The first sign is usually a history of night-time snoring; nevertheless, this is not oropharyngeal snoring but laryngeal stridor. Video polysomnography indirectly may distinguish between the two kinds of snoring [8] by examining respiratory phases, if it is inspiratory, expiratory, or both, during the snoring wave report. The pathology underlying MSA has been identified as cell loss and gliosis throughout much of the Central Nervous System. The caudate nucleus and putamen, the globus pallidus, and the substantia nigra are very involved. The pontine nuclei and the Purkinje cells of the cerebellum are also involved as well as the locus ceruleus and vestibular nuclei. The inferior olives and the dorsal motor nucleus of the vagus and pyramidal tracts are also affected. The smaller diameter fibers of the autonomic nervous system appear to be the first involved, followed by the gamma fibers and alpha fibers [1, 9]. The clinical diagnosis of MSA is difficult and there are no pathognomonic features to discriminate the Parkinsonian variant (MSA-P) from Parkinson's disease. Hence some warning signs, also called red flags, may be helpful to corroborate MSA as effective diagnosis. They are divided into six categories: early instability, rapid progression, abnormal postures, bulbar dysfunction, respiratory dysfunction, and emotional incontinence. A European MSA Study Group concluded that two out of those six red flags are highly specific with a good sensitivity for differential diagnosis when comparing MSA-P versus Parkinson Disease patients [10]. In the current case, the patient presented clinical and imaging criteria that could establish MSA as possible diagnosis. This procedure was not usual since the main complaints (respiratory insufficiency and vocal cords paralysis) are rare manifestations of MSA and only few articles relating these to MSA can be found in literature [7–9, 11, 12]. In 2006, Glass et al. [12] reported 6 cases of irregularities in respiration that may occasionally arise in an early stage of MSA and may be clues to diagnosis. Assessment of respiratory function, both during awake and asleep periods, may be helpful to successfully diagnose patients with atypical Parkinsonism, ataxia, or dysautonomia [12]. In addition, they still related that patients with MSA often have disturbances of respiratory rhythm during sleep which could explain why patients with MSA may die of respiratory insufficiency despite tracheostomy. On the other hand, cardiac arrhythmias are often noted in association with sleep-related disordered breathing, but sinus arrest is predominantly associated with obstructive sleep apnea [11]. So, this could explain why this disease has a poor overall survival. However, the mechanism underlying nocturnal sudden death in patients with MSA remains unclear. It may be explained by upper-airway obstruction, such as vocal cord abductor paralysis; an impairment of the respiratory center, such as Cheyne-Stokes respiration; or an impaired hypoxemic ventilatory response [13]. It was reported by laryngeal electromyography that patients with multiple-system atrophy and autonomic failure can present unequivocal evidence of denervation of the posterior cricoarytenoid, partial denervation in the cricopharyngeal sphincter, in the interarytenoid, cricopharyngeal sphincter and may have respiratory obstruction requiring a tracheostomy, as it happened with our patient. The cause of denervation is likely to be at the level of the nucleus ambiguous [14].

## 4. Conclusion

MSA is considered a disease with difficult diagnosis and with a bad prognosis. On clinical presentation, MSA comes with a combination of autonomic failure with Parkinsonism or cerebellar ataxia or both. Current criteria distinguish between levels of diagnostic certainty, using terms like “definite" MSA (when in the presence of anatomopathological results) and “probable” and “possible” MSA, diagnosed by clinical features [6]. As demonstrated by the current case, sometimes clinical information is unclear and other diagnostic methods acquire relevance in order to complete diagnosis, such as imaging methods [3]. Beside these facts, as reported in the published consensus of MSA in 2008 [6], the criteria for the diagnosis of MSA were simplified and now it is less cumbersome to apply in patients with probable MSA and ensure a high level of diagnostic accuracy. This disease may sometimes present itself as primary respiratory insufficiency, with initially mild motor and autonomic symptoms. Therefore, unexplained respiratory insufficiency, bilateral vocal cord paralysis, or stridor should to be taken into consideration in the diagnosis of MSA.

##  Consent

Written informed consent was obtained from the patient for publication of this case report and accompanying images. A copy of the written consent is available for review by the Editor-in-Chief of this journal.

##  Competing Interests

The authors declare that they have no competing interests.
